# Removal of Triphenylmethane Dyes by Bacterial Consortium

**DOI:** 10.1100/2012/512454

**Published:** 2012-05-01

**Authors:** Jihane Cheriaa, Monia Khaireddine, Mahmoud Rouabhia, Amina Bakhrouf

**Affiliations:** ^1^Laboratory of Analysis, Treatment, Valorisation and Environmental Pollution and Products “LR01ES16”, Faculty of Pharmacy, University of Monastir, Monastir Avicenne Street, Monastir 5000, Tunisia; ^2^Groupe de Recherche en Ecologie Buccale, Faculté de Médecine Dentaire, Université Laval 2420 rue de la Terrasse, QC, Canada G1V 0A6

## Abstract

A new consortium of four bacterial isolates (*Agrobacterium radiobacter; Bacillus spp.; Sphingomonas paucimobilis, and Aeromonas hydrophila*)-(CM-4) was used to degrade and to decolorize triphenylmethane dyes. All bacteria were isolated from activated sludge extracted from a wastewater treatment station of a dyeing industry plant. Individual bacterial isolates exhibited a remarkable color-removal capability against crystal violet (50 mg/L) and malachite green (50 mg/L) dyes within 24 h. Interestingly, the microbial consortium CM-4 shows a high decolorizing percentage for crystal violet and malachite green, respectively, 91% and 99% within 2 h. The rate of chemical oxygen demand (COD) removal increases after 24 h, reaching 61.5% and 84.2% for crystal violet and malachite green, respectively. UV-Visible absorption spectra, FTIR analysis and the inspection of bacterial cells growth indicated that color removal by the CM-4 was due to biodegradation. Evaluation of mutagenicity by using *Salmonella typhimurium* test strains, TA98 and TA100 studies revealed that the degradation of crystal violet and malachite green by CM-4 did not lead to mutagenic products. Altogether, these results demonstrated the usefulness of the bacterial consortium in the treatment of the textile dyes.

## 1. Introduction

Crystal violet (N, N, N′, N′, N′′, N′′-hexamethyl pararosaniline, CV) and malachite green (dimethylamino-4-alpha-phenylbenzylidene-4-cyclohexadiene-2, 5-ylidenedimethyl ammonium, MG) were triphenylmethane (TPM) dyes [1, 2]. They are extensively used in textile dyeing and dye-stuff manufacturing industries, as a biological stain and in printing paper [3]. The expanding use of TPM dyes in textile industries for dying nylon, polyacrylonitrile-modified nylon, wool, silk, and cotton [4–6] is alarming, given that the release of colored compounds into the environment may cause substantial ecological damage. Indeed, the presence of dye as little as 10 to 20 mg/L in water is visible and affects water transparency, which may have an impact on photosynthesis in aquatic plants and causes a part of aesthetic deterioration [2, 7]. Some of the TPM dyes are aromatic xenobiotic compounds that are widely considered to be one of the main culprits of environmental pollution [8, 9]. Therefore, chemical decolorization leads to serious problems, not only due to their color, but also because many dyes and their breakdown products may be toxic and/or mutagenic to living organisms [10–12]. Biological decolorization and degradation are an environmentally friendly and cost-competitive alternative to chemical decomposition [13].

In one hand, several studies have reported the decolorization or/and biodegradation of malachite green and crystal violet dyes by pure single microorganisms including alga, fungi, yeast, and bacteria [8, 10, 14–16]. In the other hand, other works showed the importance of achieving color treatment by a bacterial consortium [10, 17]. Nevertheless, one key to efficient dye degradation is to use broad spectrum and highly efficient dye-decolorizing microorganisms [8]. However, several physicochemical parameters affecting the degradation of dyes such as dry weight of microorganisms, pH, and the decolorization system [8, 17].

The present study aims to compare the biodegradation of two dyes having structural similarity belonging to the TPM group by a bacterial consortium under the same conditions. To reach our objective, we first isolated local bacterial strains based on their capacity to decolorize CV and MG dyes. Second, we monitored dyes biodegradation using color and COD removal, UV-Visible spectra and FTIR analyses. Finally, mutagenicity of biodegradation products was tested in the bacterial reverse mutation assay.

## 2. Materials and Methods

### 2.1. Chemicals Dyes and Media

The triphenylmethane dyes used in this study were crystal violet (C.I. 42555, prod: 34024) and malachite green (C.I. 42000, S. Nr.754). The composition of mineral agar salts medium (MSM)-agar-dye (g/L) was as following: 6 Na_2_HPO_4_, 0.5 NaCl, 3 K_2_HPO_4_, 0.1 MgSO_4_, 0.14 NH_4_Cl, 3.0 bactocasamino acids (Difco, USA), 15 Agar-agar (Fluka), and 50 mg/L of dyes [6]. The MSM-liquid medium was prepared by the following components as indicated by Moutaouakkil et al. [18]: MgSO_4_ 0.1 g/L, (NH4)_2_SO_4_ 0.6 g/L, NaCl 0.5 g/L, K_2_HPO_4_ 1.36 g/L, CaCl_2_ 0.02 g/L, MnSO_4_ 1.1 mg/L, ZnSO_4 _  0.2 mg/L, CuSO_4_ 0.2 mg/L, and FeSO_4_ 0.14 mg/L in 1000 mL of distilled water and pH was 7.5. The medium was sterilized at 120°C for 15 min. Dyes solutions, BactoTM yeast extract (Difco, USA) at 1% and glucose C_6_H_12_O_6_ (FW: 180) at 100 mM were sterilized through a 0.2 *μ*m membrane filter.

### 2.2. Effect of pH on Dyes Absorbance

Different 500 mL flasks containing 250 mL of MSM-liquid medium were prepared. The pH of each medium was adjusted to (3.5, 7.5 and 11.5) using NaOH or HCl solutions (1N). Each flask was supplemented with CV or MG dyes at 50 mg/L, mixed adequately to dissolve the dye, and used to determine dye specific absorbance. For this purpose, the analyses of spectra were monitored by spectrophotometry by using a spectrophotometer (Shimadzu UV-2401 PC model Kyoto, Japan). Experiment was realized at 25°C (*n* = 5).

### 2.3. Screening and Identification of Microorganisms

More than one hundred microbial strains were isolated from a wastewater treatment plant of regional textile industry. Firstly, a screening on solid MSM-agar-dye media was carried out according to the method used by Cheriaa and Bakhrouf [14]. Then, all plates were incubated at 30°C for 48 h. The active microorganisms were selected according to their ability to decolorize dyes by forming clear zones around colonies. Microorganisms that produced clear zones of diameter (*≈* 10 mm) were selected to get pure culture. Secondly, the four selected isolates were cultivated onto trypticasein soy agar plates and incubated overnight at 30°C. The developed colonies were submitted to Gram stain, oxidase, and catalase tests. Three strains were founded with gram-negative rods and oxidase positive, then, the identification was performed by using API 20NE Test System (bio-Mérieux, Marcy l'Etoile, France) at 37 ± 2°C for 48 h. The species were identified as (*Agrobacterium radiobacter*; *Sphingomonas paucimobilis*: *Aeromonas hydrophila*). The strain giving gram-positive Bacilli with catalase positive was identified by a biochemical Kit API 50 CH (bio-Mérieux, Marcy l'Etoile, France) which was incubated at 37 ± 2°C for 24 h [19] and the strain species was belonging to the* Bacillus spp*.

### 2.4. Bacterial Inocula

Selected strains were precultured in Luria-Bertani (LB) broth medium under agitation (rotary shaker, 120 rpm) at 37 ± 2°C overnight and the cells were collected by centrifugation at 5,000 g for 5 min. The cell pellets were washed twice with sterilized MSM and adjusted to an OD_600_ of 1.0. Each culture was tested individually for its decolorization effect in order to ensure the bacterium efficiency to decolorize dyes. Cells of individual isolates were inoculated separately onto eight flasks at 1% (v/v). Then, bacterial consortium based on four microbial strains (*Agrobacterium radiobacter*,* Bacillus spp.*, *Sphingomonas paucimobilis,* and *Aeromonas hydrophila*) (CM-4) was developed by mixing equal volumes of each individual isolate. For degradation experiments, the cells of CM-4 were inoculated into flasks at 4% (v/v) level. All experiments were conducted in the same conditions consisting of a 500-mL flask containing 250 mL MSM (pH 7.5). Culture medium was supplemented with 50 mg/L of the dye to be tested in the presence of yeast extract (0.1%) and glucose (7 mM) [14]. Flasks were incubated at 37°C, under shaking (120 rpm) in a rotary shaker. Samples were collected at different time points (2, 4, 6, 8, and 24 h) to determine the dyes decolorization.

### 2.5. Decolorization Assay

For determination of CV and MG color removal, 5 mL aliquots of the culture were sampled at different culture periods (2, 4, 6, 8, and 24 h), centrifuged at 6,000 g for 10 min to eliminate the bacterial cells, and the supernatant was examined by spectrophotometry (Spectrophotometer, Shimadzu UV-2401 PC model, Kyoto, Japan) at the (*λ*max) of 590 nm and 618 nm, respectively, for CV and MG [20]. The percentage of decolorization was calculated as following: decolorization (%) = [(Absorbance at *t*_0_) − (Absorbance at *t*_1_)]/(Absorbance at *t*_0_) × 100]. Dye elimination was investigated with each selected bacterial strain and with the bacterial consortium.

### 2.6. COD Removal of CM-4

Potassium dichromate oxidation was analyzed using culture supernatants. Aliquots were taken at different culture periods (2, 4, 6, 8, and 24 h), then centrifuged at 4,000 g for 15 min. The supernatant was mixed with a commercial solution Hach medium in tubes (150–1500 mg/L). Then, chemical oxygen demand (COD) value was directly determined by using HACH 2000 spectrophotometer. The percentage of COD removal was calculated as following: COD  (%) = [(COD  at  *t*_0_)−(COD  at  *t*_1_)]/(COD  at  *t*_0_) × 100]. The percentage determination of COD values and color removal were performed at the same time.

### 2.7. Fourier Transformation Infrared Spectroscopy (FTIR) Analysis

A volume of 100 mL was taken from each flask at 0 h before biological treatment and at 2 h after biodegradation by the bacterial consortium, then centrifuged at 6,000 g for 10 min and the supernatant was lyophilized. A portion of 1.5 milligram of each lyophilized samples was mixed with 200 mg of potassium bromide and discs were obtained under vacuum pressure at 250 atm for 10 min. Discs were examined in a FTIR spectrophotometer (FTIR-8400, Shimadzu, Japan) over the wavelength (4000–500 cm^−1^) with a resolution of 1 cm^−1^.

### 2.8. Mutagenicity (Ames Test)

In this work, mutagenicity was tested at two concentrations 50 and 25 mg/L of dyes in mineral salts medium (MSM-liquid), before and after biological treatment by CM-4. Samples of 100 mL were taken after 24 hours of biodegradation of CV and MG, then cells were removed, by centrifugation at 15,000 × g for 20 min [1] and the supernatant was filtered through a 0.45 *μ*m filter (Millipore, Sartorius Minisart CE 0297, Germany). Thereafter, the supernatant obtained and the MSM-liquid dyes solutions were lyophilized. The investigation was performed with the following concentrations at 250 and 500 *μ*g/plate obtained by lyophilization and were added to a minimal medium soft-agar overlay containing 0.1 mL of *Salmonella typhimurium* test strains, TA98, and TA100 as used by Schneider et al. [21]. Each dose was tested in duplicate. Plates were incubated at 37°C for 48 h before counting revertant colonies. mutagenic ratio was calculated as following: mutagenic ratio (MR) = mean revertants/mean spontaneous revertants as indicated by Azizan and Blevins [22].

## 3. Results and Discussion

### 3.1. Effect of pH on Dyes

**Figure 1:**
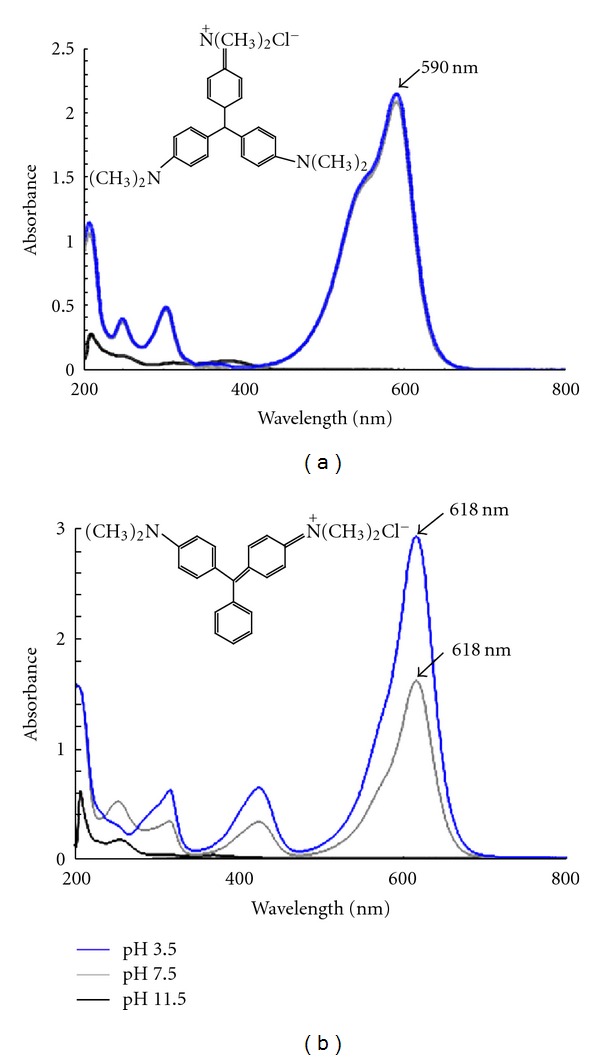
Chemical structure of crystal violet (a) and malachite green (b) dyes used in the present study and their spectra profiles obtained at the following pH (3.5, 7.5, and 11.5), in mineral salts medium at concentration 50 mg/L, the experience was realized at 35°C.

UV-visible spectra of crystal violet (CV) and malachite green (MG) at concentration (50 mg/L) in visible region exhibit a main peak with a maximum absorbance at *λ*max at (590 nm and 618 nm) (Figures 1(a) and 1(b)). It is interesting to note that both CV and MG spectra were not affected by the variation pH (at 3.5 and at 7.5), and critical peak persists at the same wavelength. Nevertheless, the absorbance amplitude of critical peak at 618 nm for MG decreases at pH (7.5), thus MG color intensity may be affected at high pH level. Moreover, the color of CV and MG disappeared completely from MSM dyes solution in alkali condition (Figure 1) at pH 11. 5, thus pH has an effect on the color stability. Our data are in accordance with those reported previously by Mahmoud et al. [23]. Triphenylmethane dyes were organic compound soluble in water, adding an OH^−^ ion in central carbon converts the cation into carbinol base nonresonant (Figure 1(a)). Consequently, the conjugation between the benzene nuclei was then interrupted and the molecule becomes colorless [24]. Indeed, some procedures of treating industrial textiles wastewater were based on chemical processes to reduce color as a pollutant factor [25]. However, for MG dye the absorbance decreases when the pH medium was 7.5, this result encourages biological treatment of MG because favorable pH for dye decolorization by bacterial strains ranged from 7 to 8 with an optimal pH being 7 [10].

### 3.2. Decolorization and COD Removal

**Figure 2:**
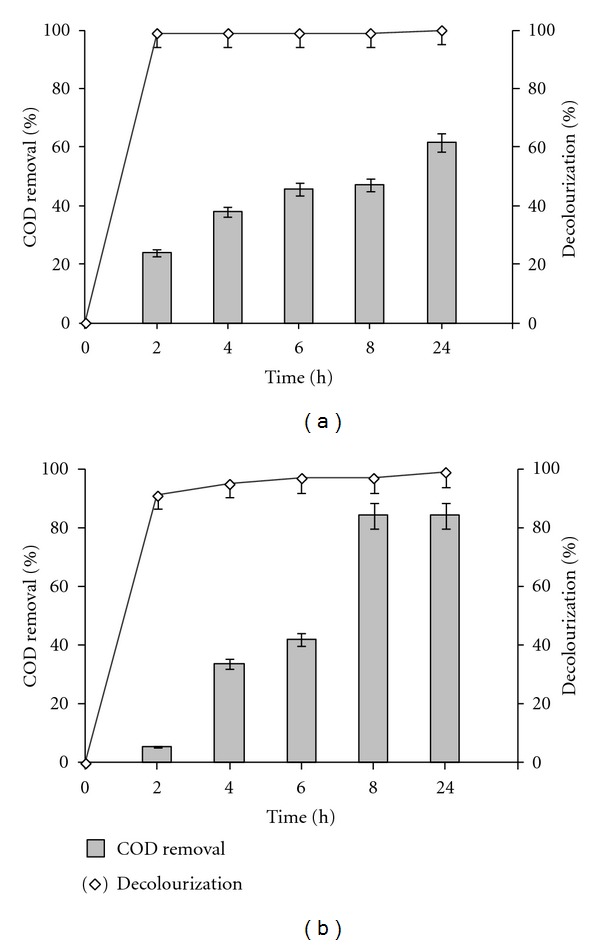
Decolorization and COD removal dyes, crystal violet (a), and malachite green (b), obtained by a consortium CM-4 in MSM medium supplemented with 0.1% yeast extract and 7 mM glucose at 35°C during 24 h, under agitation conditions (120 rpm).

Pure culture of isolates strains tested individually can decolorize crystal violet (CV) and malachite green (MG) with an efficient rate. Greater decolorization was reached within 24 h as indicated in Table 1. Interestingly, decolorization by using bacterial consortium (CM-4) was more rapid, (99%) for MG and (91%) for CV within 2 h (Figures 2(a) and 2(b)). Using microbial consortium may increase the decolorization rate of dyes due to synergistic reaction between microorganisms [20, 26]. High percentages of decolorization (CV and MG) were obtained reaching 100% within 10 h by a single bacteria *Sphingomonas paucimobilis* [14]. Indeed, the addition of three other strains (*Agrobacterium radiobacter*; *Bacillus spp*.; *Aeromonas hydrophila*); enhanced the decolorization to reach 100% within only 2 hours (Table 1). Decolorization and biodegradation of different dyes may be reached by different microorganisms including alga, fungi, yeast, and bacteria [10, 14, 15].

Furthermore, the difference between decolorization rates may be allotted to the effect of the pH medium (7.5) to reduce MG color (Figure 1(b)). The percentage of decolorization could be due to the effect of biological treatment and also to the physicochemical parameters including pH. For crystal violet, efficiency of decolorization by the consortium does not cause the increase of COD removal. This was about 61.5% for CV and 84.2% for MG, within 24 h of incubation (Figures 2(a) and 2(b)). However, there is no exact correlation between the percentage of decolorization and COD removal. This observation confirms those previously reported by Mohana et al. [27] arguing that a high decolorization percentage does not mean systematically a high value of COD removal. The rise of COD removal percentage may be due to the decomposition of organics dyes by a bacterial consortium CM-4. Indeed, color removal from industrial textile wastewater does not translate a low value into COD. Such hypothesis needs further investigations.

### 3.3. UV-Visible Spectra of Decolorized Dyes

**Figure 3:**
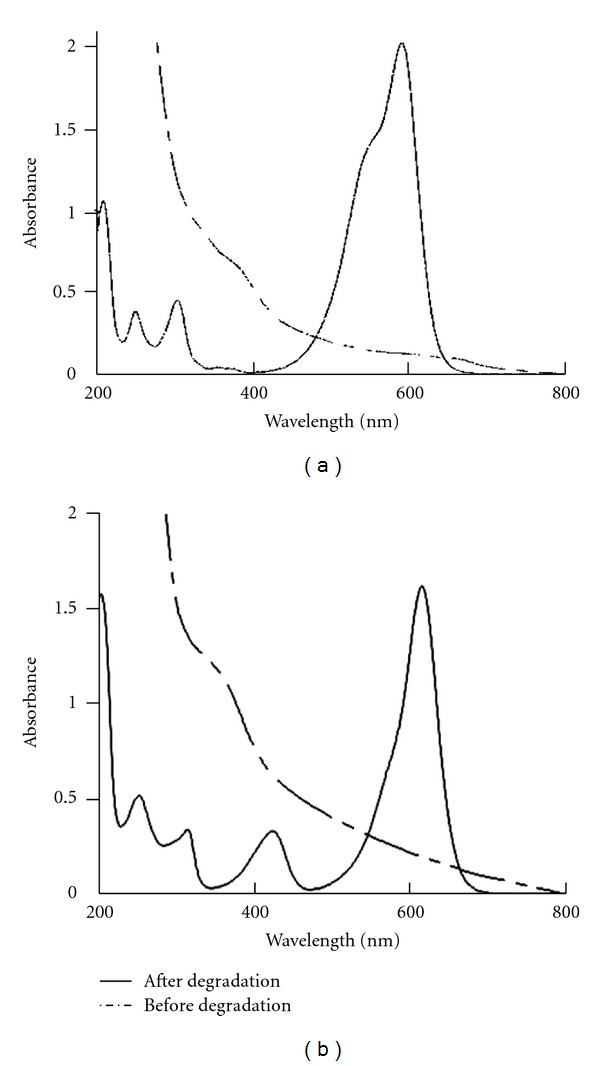
UV-visible spectra of crystal violet (a) and malachite green (b) at 0 h (control) and after 2 h, decolorized by CM-4 in mineral salts medium water (pH 7.5). The experience was performed by a bacterial consortium CM-4 with dyes at concentrations (50 mg/L) supplemented by yeast extract (0.1%) and glucose (7 mM), at 35°C, under shaking conditions (120 rpm).

The results of the UV-Visible spectra showed that peaks at *λ*max reached at 0 h decreased significantly until disappearing completely within 2 h (Figure 3). No new peak appeared in visible range; however, an extra absorbance was examined in decolorized solution for CV and MG, with the appearance of peaks at 220 and at 260 nm. The absorbance between (200–300 nm) in UV region for the tested dyes was highly similar (Figures 3(a) and 3(b)). This absorbance was probably a result of identical metabolites or/and degraded fragments obtained after degradation of dyes molecules. Indeed, Jang et al. [28] obtained the same results when analyzing UV-visible spectrum of crystal violet. The disappearance of crystal violet, which has maximum absorbance at 590 nm, and the concomitant appearance of a product with an absorbance maximum at 260 nm characterize the authentic leucocrystal violet [28]. Moreover, the examination of microbial biomass after complete decolorization remains colorless, confirming that the biodegradation of the dyes was achieved by bacterial cells. This observation is in favor of biodegradation of CV and MG dyes by CM-4. Indeed several studies [1, 29, 30] attributed decolorization to the biodegradation when the major visible light absorbance peaks of the dyes disappeared, but the cells causing degradation persist colorless. Biodegradation by CM-4 of CV and MG were similar, this is may be explained by the strong resemblance in their chemical structure. The reduction of crystal violet and malachite green to its leuco form is under the control of the triphenylmethane reductase (TMR). However, the most efficient TMR substrate appeared to be malachite green, because it exhibited favorable structural features when modeled with the ternary complex [28]. Crystal violet was a less favorable TMR substrate than malachite green, perhaps because of the additional dimethylamino group [8]. Indeed, our results (Table 1) and (Figure 2) showed that MG decolorization was higher than CV; therefore, this activity depends on the chemical structure of dyes.

### 3.4. FT-IR Spectra of Decolorized Dyes

**Figure 4:**
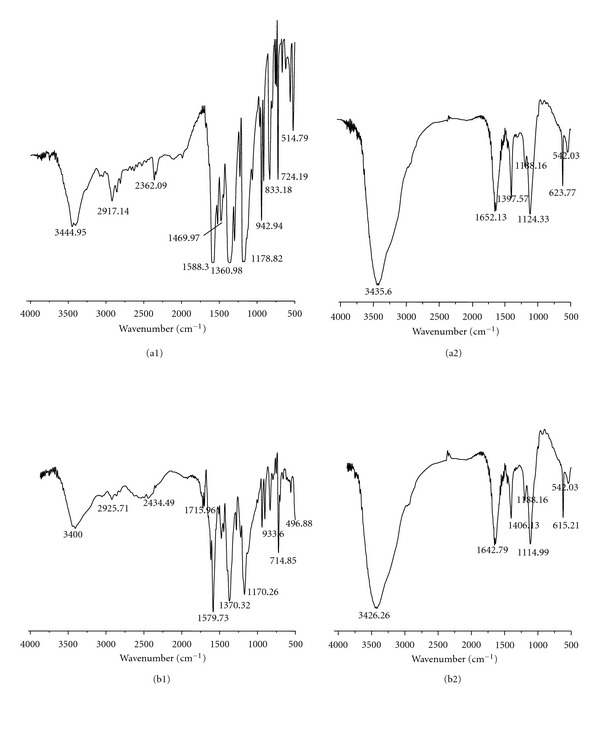
FT-IR spectra of crystal violet and malachite green (a1) and (b1) at 0 h (control), respectively. The spectra (a2) and (b2), for CV and MG were obtained after 2 h of degradation by a consortium CM-4, the experiment was realized at the same conditions.

Our results showed remarkable variations in the fingerprint region ranged from 1500 to 500 cm^−1^, of the FT-IR spectroscopy as indicated in Figures 4(a1) and 4(a2) and in Figures 4(b1) and 4(b2), for CV and MG, respectively. According to the FTIR spectra, the assignments of the major infrared bands obtained with each dye at 0 h and 2 h incubation with CM-4 were listed in Table 2.

For CV, the most specific peaks at 0 h were monosubstituted and paradisubstituted benzene rings which is supportive to the peak at 1588.30 cm^−1^ corresponding to the C=C stretching of the benzene ring. Spectrum shows also a peak at 1178.82 cm^−1^ corresponds to the C–N stretching vibrations and a peak at 2917.14 cm^−1^ for C–H stretching of asymmetric CH_3_ group. The peak observed in region between 2300 and 2400 cm^−1^ corresponding to a symmetric and asymmetric stretching of tertiary amine salt. The peak for the C–N stretch of aromatic tertiary amine was observed at 1360.98 cm^−1^. After, biodegradation by CM-4 (Figures 4(a2)), the peak at 1652.13 cm^−1^ in the region of 1680–1620 cm^−1^ can be attributed to C=C stretch, which underlined a degradation of aromatic ring.

For MG, specific peaks between 1500 and 500 cm^−1^ correspond to the monosubstituted and paradisubstituted benzene rings which is supporting the peak at 1579.73 cm^−1^ corresponding to the C=C stretching of the benzene ring. Spectrum also shows a peak at 1170.32 cm^−1^ corresponding to the C-N stretching vibrations and a peak at 2925.71 cm^−1^ for C–H stretching of asymmetric CH_3_ group. The two bands located initially between 2300 and 2400 cm^−1^ correspond to the symmetric and asymmetric vibration of salt tertiary amine which disappeared after treatment and imply that the tertiary amines of MG were transformed. Moreover, the absence of peaks between (850–670 cm^−1^) supports the total disappearance of aromatic rings for CV and MG, Figures 4(a2) and 4(b2), respectively. According to Coates [31], more than one peak obtained in region of the C–H bending vibrations (850–670 cm^−1^) can support the presence of an aromatic structure. In addition, FT-IR spectra of dyes show the presence of two bands between (3200 and 3500 cm^−1^) allotted to N–H stretching vibrations of primary amines. These results prove the degradation action of bacterial consortium CM-4 and the mechanism of cleavage of chemical structure of dyes for CV and MG that seem identical under aerobic conditions.

### 3.5. Mutagenicity Test

The tests of mutagenicity were carried out on the extracellular liquid after biodegradation of dyes within 2 h. The concentrations of crystal violet and malachite green tested (250 and 500 *μ*g per plate) do not show a mutagen effect on strain's TA98 and TA100 as indicated in Table 3. Therefore, the metabolites and/or chemical compounds obtained by a biodegraded bacterial consortium (CM-4), did not present risks of mutagenicity. In fact, bacterial consortium CM-4 has a significant value in the remediation of the effluents charged in dyes belonging to the triphenylmethane class. Similar results were reported by Schneider et al. [21] and Clemmensen et al. [32] with malachite green. Further studies are needed to confirm the absence of mutagenic effect of the dyes.

## 4. Conclusion

This work shows a high potential of bacterial consortium to decolorize triphenylmethane dyes in conjunction with pH. The mechanisms of biodegradation for CV and MG by the CM-4 were similar; this indicated that decolorization activity was independent on the chemical structure of the dyes but attributed to the nature of mechanisms involved in bacterial strain. Nevertheless, a special opportunity for efficient dye degradation was to use various microorganisms belonging to different taxonomic groups having highly efficient dyedecolorizing.

## Figures and Tables

**Table 1:** Decolorization of dyes by individual pure culture of isolated bacterial strain and by a bacterial consortium.

Dyes	Time (h)	S_1_	S_2_	S_3_	S_4_	S_5_
CV	2	7 ± 0.2	5 ± 0.3	8 ± 0.2	3 ± 0.1	99 ± 0.3
	4	39 ± 0.4	25 ± 0.3	44 ± 0.3	31 ± 0.5	99 ± 0.2
	6	61 ± 0.3	39 ± 0.4	87 ± 0.1	43 ± 0.1	99 ± 0.2
	8	77 ± 0.2	45 ± 0.5	92 ± 0.2	50 ± 0.2	99 ± 0.1
	24	85 ± 0.7	71 ± 0.2	92 ± 0.3	64 ± 0.9	100 ± 0.1

MG	2	0.6 ± 0.3	0.1 ± 0.4	0.8 ± 0.4	0.4 ± 0.2	91 ± 0.3
	4	20 ± 0.3	12 ± 0.3	29 ± 0.2	16 ± 0.3	95 ± 0.1
	6	63 ± 0.2	37 ± 0.2	68 ± 0.3	40 ± 0.4	97 ± 0.3
	8	89 ± 0.4	60 ± 0.5	96 ± 0.2	64 ± 0.2	97 ± 0.4
	24	94 ± 0.3	97 ± 0.1	93 ± 0.4	94 ± 0.1	99 ± 0.1

Percentages of decolorization of crystal violet (CV) and malachite green (MG) were calculated after (2, 4, 6, 8, and 24 hours) culture at 35°C under agitation conditions, with yeast extract (0.1%) and glucose (7 mM) as an additional carbon source. Dyes were present at a concentration 50 mg/L. S_1_, *Agrobacterium radiobacter*; S_2_, *Bacillus spp.*; S_3_, *Sphingomonas paucimobilis*; S_4_, *Aeromonas hydrophila*; S_5_, bacterial consortium (CM-4).

**Table 2:** Assignments of the FTIR spectral peaks of crystal violet (CV) and malachite green (MG).

	Wavelength (cm^−1^)	Vibration	Functional or component
CV control at (0 h)	3444.93	O–H stretch	Hydroxyl groups and water
	1588.30	C=C stretch	Aromatic ring
	1178.82	C–N stretch	Tertiary amine
	2917.14	C–H stretch	CH_3_ group
	1360.98	C–N stretch	Aromatic tertiary amine

CV at (2 h)	3435.6	O–H stretch	Hydroxyl groups, phenols, and water
		N–H stretch	Aliphatic primary amine
	1652.13	C=C	Alkenyl C=C stretch
	1124.33	C–N	Aliphatic amine

MG control at (0 h)	3400	O–H stretch	Hydroxyl groups, and water
	1579.73	C=C stretch	Aromatic ring
	1170.26	C–N stretch	Tertiary amine
	2925.71	C–H stretch	CH_3_ group
	1370.32	C–N stretch	Aromatic tertiary amine

MG at (2 h)	3426.26	O–H stretch	Hydroxyl groups, phenols and water
		N–H stretch	Aliphatic primary amine
	1642.79	C=C	Alkenyl C=C stretch
	1114.99	C–N	Aliphatic amine

**Table 3:** Response of triphenylmethane dyes before and after treatment by a new bacterial consortium CM-4 in *Salmonella typhimurium* (TA98 and TA100) without metabolic activation.

*S. typhimurium* strains				
	TA98	TA100	MR for TA98	MR for TA100

Mean revertants per plate^a^				

SR	17.5 ± 3.5	28 ± 4.2		

Samples untreated				

CV^1^	22.5 ± 3.5	41.5 ± 2.1	1.2	1.4
CV^2^	24.5 ± 2.1	44.5 ± 6.3	1.4	1.5
MG^1^	12 ± 2.8	28.5 ± 4.9	0.6	1.0
MG^2^	17.5 ± 2.1	34.5 ± 4.9	1.0	1.2

Samples treated by CM-4				

CV^1^	20.5 ± 2.1	29 ± 1.4	1.1	1.0
CV^2^	14.5 ± 2.1	17 ± 1.4	0.8	1.0
MG^1^	14 ± 2.8	36 ± 1.4	0.8	1.2
MG^2^	9.5 ± 2.1	26.5 ± 2.1	0.5	0.9

CV, crystal violet; MG, malachite green; *S.*,* Salmonella*; SR, spontaneous revertants; ^ 1^, 500 *μ*g per plate; ^2^, 250 *μ*g per plate; ^a^values obtained from duplicate plates; MR, mutagenic ratio.

## References

[1] Sun-Young A, Sang-Ki M, Yong-Lark CI-H, Young-Su C, Cherol-Ho K, Young-Choon L (2002). Decolorization of triphenylmethane and azo dyes by *Citrobacter sp.*. *Biotechnology Letters*.

[2] Kavita V, Ramesh CK, Rajendra KS (1995). Decolourization of Triphenylmethane Dyes by the Bird's Nest Fungus *Cyathus bulleri*. *Current of Microbiology*.

[3] Kumar RS, Banerjee UC (1999). Decolorization of triphenylmethane dyes and textile and dye-stuff effluent by *Kurthia sp.*. *Enzyme and Microbial Technology*.

[4] Daneshvar N, Ayazloo M, Khataee AR, Pourhassan M (2007). Biological decolorization of dye solution containing Malachite Green by microalgae *Cosmarium sp.*. *Bioresource Technology*.

[5] Parshetti G, Kalme S, Saratale G, Govindwar S (2006). Biodegradation of malachite green by *Kocuria rosea* MTCC 1532. *Acta Chimica Slovenica*.

[6] Gregory P, Kroschwitz JI (1993). Dyes and dye intermediates. *Encyclopedia of Chemical Technology*.

[7] Alinsafi A, da Motta M, Le Bonté S, Pons MN, Benhammou A (2006). Effect of variability on the treatment of textile dyeing wastewater by activated sludge. *Dyes and Pigments*.

[8] Kim MH, Kim Y, Park HJ (2008). Structural insight into bioremediation of triphenylmethane dyes by *Citrobacter sp.* triphenylmethane reductase. *The Journal of Biological Chemistry*.

[9] Sharma DK, Saini HS, Singh M, Chimni SS, Chadha BS (2004). Biodegradation of acid blue-15, a textile dye, by an up-flow immobilized cell bioreactor. *Journal of Industrial Microbiology and Biotechnology*.

[10] Ali H (2010). “Biodegradation of synthetic dyes”, A review. *Water, Air and Soil Pollution*.

[11] Li LT, Hong Q, Yan X, Fang GH, Shinawar WA, Li SP (2009). Isolation of a malachite green-degrading *Pseudomonas* sp. MDB-1 strain and cloning of the tmr2 gene. *Biodegradation*.

[12] Weisburger JH (2002). Comments on the history and importance of aromatic and heterocyclic amines in public health. *Mutation Research*.

[13] Robinson T, McMullan G, Marchant R, Nigam P (2001). Remediation of dyes in textile effluent: a critical review on current treatment technologies with a proposed alternative. *Bioresource Technology*.

[14] Cheriaa J, Bakhrouf A (2009). Triphenylmethanes, malachite green and crystal violet dyes decolourisation by *Sphingomonas paucimobilis*. *Annals of Microbiology*.

[15] Cheriaa J, Bettaieb F, Denden I, Bakhrouf A (2009). Characterization of new algae isolated from textile wastewater plant. *Journal of Food, Agriculture and Environment*.

[16] Arroyo-Figueroa G, Ruiz-Aguilar GML, López-Martínez L, González-Sánchez G, Cuevas-Rodríguez G, Rodríguez-Vázquez R (2011). Treatment of a textile effluent from dyeing with cochineal extracts using *Trametes versicolor* fungus. *The Scientific World Journal*.

[17] Sharma DK, Saini HS, Singh M, Chimni SS, Chadha BS (2004). Isolation and characterization of microorganisms capable of decolorizing various triphenylmethane dyes. *Journal of Basic Microbiology*.

[18] Moutaouakkil A, Zeroual Y, Blaghen M Decolourisation and biodegradation of toxic azo-dye Methyl Red by *Enterobacter agglomerans*.

[19] Gacitúa A S, Valiente CF, Díaz KP, Hernández JC, Uribe MM, Sanfuentes EV (2009). Identification and biological characterization of isolates with activity inhibitive against *Macrophomina phaseolina *(Tassi) goid. *Chilean Journal of Agricultural Research*.

[20] Manjinder SK, Harvinder SS, Deepak KS, Bhupinder SC, Swapandeep SC (2005). Decolorization of various azo dyes by bacterial consortium. *Dyes and Pigments*.

[21] Schneider K, Hafner C, Jäger I (2004). Mutagenicity of textile dye products. *Journal of Applied Toxicology*.

[22] Azizan A, Blevins RD (1995). Mutagenicity and antimutagenicity testing of six chemicals associated with the pungent properties of specific spices as revealed by the Ames *Salmonella*/microsomal assay. *Archives of Environmental Contamination and Toxicology*.

[23] Mahmoud AS, Ghaly AE, Brooks MS (2007). Removal of dye from textile wastewater using plant oils under different pH and temperature conditions. *American Journal of Environmental Sciences*.

[24] García-Río L, Leis JR, Mejuto JC, Navarro-Vázquez A, Pérez-Juste J, Rodriguez-Dafonte P (2004). Basic hydrolysis of crystal violet in *β*-cyclodextrin/surfactant mixed systems. *Langmuir*.

[25] Pourrezaei P, Afzal A, Ding N (2010). Physico-chemical processes-review. *Water Environment Research*.

[26] He F, Hu W, Li Y (2004). Biodegradation mechanisms and kinetics of azo dye 4BS by a microbial consortium. *Chemosphere*.

[27] Mohana S, Desai C, Madamwar D (2007). Biodegradation and decolourization of anaerobically treated distillery spent wash by a novel bacterial consortium. *Bioresource Technology*.

[28] Jang MS, Lee YM, Kim CH (2005). Triphenylmethane reductase from *Citrobacter sp.* strain KCTC 18061P: purification, characterization, gene cloning, and overexpression of a functional protein in *Escherichia coli*. *Applied and Environmental Microbiology*.

[29] Chen KC, Wu JY, Liou DJ, Hwang SCJ (2003). Decolorization of the textile dyes by newly isolated bacterial strains. *Biotechnology*.

[30] Daneshvar N, Khataee AR, Rasoulifard MH, Pourhassan M (2006). Biodegradation of dye solution containing malachite green: optimization of effective parameters using Taguchi method. *Journal of Hazardous Materials*.

[31] Coates J, Meyers RA (2000). Interpretation of infrared spectra. A practical approch. *Encyclopedia of Analytical Chemistry*.

[32] Clemmensen S, Jøgrn CJ, Niels JJ, Otto M, Preben O, Gunna W (1984). Toxicological studies on malachite green: a triphenylmethane dye. *Archives of Toxicology*.

